# Nutrient loading and farm characteristics of giant gourami fish aquaculture systems in Lake Maninjau, Indonesia: basic knowledge of production performance

**DOI:** 10.12688/f1000research.52613.1

**Published:** 2021-05-12

**Authors:** Hafrijal Syandri, Azrita Azrita, Eni Sumiarsih, Elfiondri undefined

**Affiliations:** 1 Department of Aquaculture, Faculty of Fisheries and Marine Science, Universitas Bung Hatta, Padang, West Sumatera, 25133, Indonesia; 2 Department of Biology Education, Faculty of Education, Universitas Bung Hatta, Padang, West Sumatera, 25133, Indonesia; 3 Department of Aquaculture, Faculty of Fisheries and Marine Science, Universitas Riau, Pekanbaru, 28293, Indonesia; 4 Faculty of Humanities, Universitas Bung Hatta, Padang, West Sumatera, 25133, Indonesia

**Keywords:** Lake Maninjau, giant gourami culture, floating cage aquaculture, nutrient loading, farm characteristics.

## Abstract

Background

Aquaculture systems for giant gourami, *Osphronemus goramy* Lacepède (1801), have significantly improved fish production yields and food security in Indonesia. However, these systems also cause serious problems in terms of eutrophication in waterbodies. This study analysed the nutrient loading and farm characteristics of giant gourami in floating cages in Lake Maninjau.

Method

A total of 20 floating cages were used to record these nutrients in feed supply, female and male juvenile fish, dead fish and harvested fish to estimate nutrient loading. Data on the harvested fish, production cycle, stock number and cage capacity were used to estimate the stocking density, feeding rate, feed efficiency, and net fish yield, and the relationship between feed supply and nutrient loading and farm characteristics was analysed by least squares regression methods.

Results

A total of 20 floating cages released nutrients into waterbodies at an average rate of 236.27±60.44 kg/cycle for C, 84.52±20.86 kg/cycle for N and 8.70±3.63 kg/cycle for P. On average, fish production for each floating cage (±SD) was 1226±282 kg wet weight/cycle, and the net fish yield was 12.63±2.82 kg/m ^3^/cycle. Survival rates ranged from 86.33 to 95.27%/cycle. The production cycles varied from 160 to 175 days with feed conversion ratios between 1.60 and 1.75, feed conversion efficiencies were between 0.58 and 0.63. The production parameters that had strong relationships with the net fish yield were feed supply ( *r* ^2^=0.960), stocking rates ( *r* ^2^=0.924) and feeding rates ( *r* ^2^=0.961). In contrast, the length of the production cycle was not strongly related to the net fish yield ( *r* ^2^=0.187).

Conclusion

Nutrient loading from the supplied feed was greater than that from the harvested fish, juvenile fish and dead fish. Increasing the net fish yield in floating cages was better predicted by the stocking densities and feeding levels than by the other factors.

## Introduction

Fish are a source of protein, lipids, carbohydrates, vitamins and essential minerals. ^1^^–^^3^ Therefore, fisheries production is very important to increasing food security ^3^^,^^4^ through capture fisheries and aquaculture sectors. ^5^ To increase the global production of aquaculture, freshwater can be provided in a variety of aquaculture systems, such as freshwater ponds, tanks and floating cages. ^6^^–^^9^

Cage aquaculture is expanding in tropical lakes and has been ongoing for a long time. ^9^^–^^11^ Lake Maninjau in Indonesia has used cage aquaculture since 1992 (Nazarudin-Sepakat Aquaculture’s farm manager, pers. comm.). Some authors have reported that the dominant species being cultured in tropical lakes is tilapia, and commercial feed pellets are used. ^9^^,^^11^^–^^15^ In the past five years, fish farmers in Lake Maninjau have also conducted giant gourami fish farming activities in floating cages with commercial feed pellets because it is an economically important species for food security in Indonesia, and most of the giant gourami that have been consumed for decades have been produced by aquaculture in freshwater ponds. ^7^^,^^16^^,^^17^

Environmental impacts of tilapia aquaculture operations that have been recorded in tropical lakes have also been reported in Lake Malawi, ^11^ Lake Taihu, ^18^ Lake Victoria, ^11^^,^^15^^,^^19^ and Lake Kariba. ^9^ In contrast, Syandri *et al.* ^20^ reported that in a small lake, *i.e.*, Lake Maninjau in Indonesia, tilapia aquaculture is approximately 17 km long by 8 km wide and has mean and maximum depths of approximately 112 and 178 m, respectively. Many studies have been carried out to evaluate nutrient loading, such as C, N and P loadings, and the growth performance of tilapia farms in lakes and reservoirs. ^11^^,^^12^^,^^18^ However, no data are available for nutrient waste loads from feed, juvenile fish, dead fish and harvested fish, including data on the characteristics of farming giant gourami in floating cages, such as stocking density, total stock weight, feed conversion ratio, production cycle, harvest size, feeding level and specific growth rate. To address these issues, the present study was conducted to evaluate the C, N and P nutrient loads of giant gourami fish in floating cages and the operational characteristics to determine the relationship between production and cultivation efficiency to provide basic knowledge about production performance for the future.

## Methods

### Ethical considerations

In the present study, no permits from the Government of the Republic of Indonesia were needed to record data on feed supply, initial weight, stocking density, fish production, fish mortality and production cycle of giant gourami in 20 floating cages in Lake Maninjau from 2019 to 2020. The study included collecting sediment and fish and killing as many as three giant gourami in each floating cage to analyse the chemical composition of carbon, nitrogen and phosphorus from the carcasses. This research was recommended by the Research and Community Service Universitas Bung Hatta with sponsorship from the Indonesian Education Management Institution, Ministry of Finance Republic of Indonesia, through a competitive grant called Productive Innovative Research 2019 with contract number PRJ-99/LPDP/2019. Ethical approval was granted by the Ethics Commission for Research and Community service at Universitas Bung Hatta (098/LPPM/Hatta/X-2019).

### Study area

The research was conducted in Lake Maninjau, located in the Agam District, West Sumatera Province, Indonesia, at an altitude of 463 m above sea level with a surface area of 97.37 km ^2^, a water volume of 10.4 km ^3^, a water retention time of 24.5 years, and a catchment area of 13.26 km ^2^. Since 1973, lake water has been used for electric power generation with a capacity of 64 MW, and starting in 1992, the lake has also been used floating-cage fish farming activities.

### Study design

A total of 200 floating cages using for giant gourami aquaculture by fish farmers in Lake Maninjau (Nazaruddin-Sepakat Aquaculture farm manager, personal communication). A total of 20 floating cages for giant gourami culture were used as samples. The sample was determined by simple random sampling using an ordinal method. ^21^ The data recorded were stock size (g), stock number (fish), total stock weight (kg), mortality (fish), feed supply (kg), total harvest weight (kg) and production cycle (days). Each floating cage had a capacity of 75 m ^3^ (5×5×3 m) and was constructed using a 10 mm mesh sieve. Each floating cage was combined with other resources, such as a buoy, a feeding lodge and cage pathways.

### Nutrient analysis

The chemical compositions carbon (C), nitrogen (N) and phosphorus (P) of the feed, fish and faeces were analysed. For the feed nutrient analysis, the feed samples were floating commercial feed (pelleted). The approximate composition of the feed was 12% moisture, 29% crude protein, 6% crude lipid, 12% crude fibre and 6% crude ash. The fish were sampled from 10 floating cages (3 fish/cage) that were cultured for 150 days, and the fish weighed between 235 and 250 g/fish. Carbon (C) and nitrogen (N) concentrations (as % of dry weight) of the feed and fish were determined by the standard methods of the Association of Official Analytical Chemists. ^22^ The phosphorus (P) concentrations were determined using a spectrophotometer (Shimadzu UV-160 UV160 UV-Vis-NIR Spectrophotometer in Hayward, CA, USA) and the molybdate–ascorbic acid method indicated by the Association of Official Analytical Chemists ^22^ at the Chemistry Laboratory of Universitas Bung Hatta Padang. To complement the data, we also analysed the waste material of cultured giant gourami fish collected with traps under the floating cages. To collect the faeces, ten giant gourami were kept for 3 days in an aquarium with a capacity of 0.48 m ^3^ (2×0.6×0.4 m), and then, the faeces were deposited on the bottom of the aquarium. Furthermore, the deposited faeces were sucked into a clean bowl and dried. Waste material and faeces were analysed by the AOAC method. ^22^

### Estimation of nutrient loading and farm characteristics

The C, N and P loadings from feed, juvenile fish, dead fish and harvested fish were estimated according to the method described by ^23^. The following parameters with their corresponding equations were analysed:

*C* (loss, kg) = ( *F* × *C*_DF_ + *J* × *C*_DJ_) − ( *H* × *C*_DH_ + *M* × *C*_DM_)

*N* (loss, kg) = ( *F* × *N*_DF_ + *J* × *N*_DJ_) − ( *H* × *N*_DH_ + *M* × *N*_DM_)

*P* (loss, kg) = ( *F* × *P*_DF_ + *J* × *P*_DJ_) − ( *H* × *P*_DH_ + *M* × *P*_DM_)

where *F*, *J*, *H* and *M* are the dry weight (kg) of the supplied feed, stocked juvenile fish, harvested fish and total dead fish in floating cages, respectively. The data were recorded at the end of each production cycle from the 20 floating cages. *C*_DF_, *C*_DJ_, *C*_DH_ and *C*_DM_ are the carbon contents in dry feed (DF), dry juvenile (DJ), dry harvest (DH) and dry mortality (DM), respectively.

The farm characteristic parameters were analysed using the following formulas:

Specific growth rate (%/day)= $\frac{\left(\mathrm{Log}\;\mathrm{harvests}\;\mathrm{weight}-\mathrm{Log}\;\mathrm{stock}\;\mathrm{weight}\right)}{\mathrm{Culture}\;\mathrm{days}}\times 100$

Gross fish yield (kg/m ^3^) = $\frac{\left(\mathrm{Total}\;\mathrm{harvest}\;\mathrm{number}\;\mathrm{in}\;\mathrm{individual}\times \mathrm{average}\;\mathrm{final}\;\mathrm{fish}\;\mathrm{weight}\;\mathrm{in}\;\mathrm{kg}\right)}{\mathrm{Cage}\;\mathrm{capacity}}$

Net fish yield (kg/m ^3^) = $\frac{\left(\mathrm{Total}\;\mathrm{number}\;\mathrm{of}\;\mathrm{fish}\;\mathrm{harvest}\;\mathrm{in}\;\mathrm{kg}-\mathrm{total}\;\mathrm{stock}\;\mathrm{weight}\;\mathrm{in}\;\mathrm{kg}\right)}{\mathrm{Cage}\;\mathrm{capacity}}$

Feed conversion ratio (FCR) = $\frac{\mathrm{Feed}\;\mathrm{supply}\;\mathrm{in}\;\mathrm{kg}}{\mathrm{Total}\;\mathrm{harvest}\;\mathrm{weight}\;\mathrm{in}\;\mathrm{kg}}$

Feed conversion efficiency (FCE) = $\frac{1}{\mathrm{Feed}\;\mathrm{conversion}\;\mathrm{ratio}}$

Feeding rate (%) = $\frac{\mathrm{Average}\;\mathrm{weight}\;\mathrm{gain}\;\mathrm{per}\;\mathrm{day}\;\mathrm{in}\;\mathrm{kg}}{\mathrm{Mean}\;\mathrm{harvests}\;\mathrm{size}\;\mathrm{in}\;\text{g}}\times 100$

Survival rate (%) = $\frac{\mathrm{Total}\;\mathrm{number}\;\mathrm{of}\;\mathrm{fish}\;\mathrm{harvested}}{\mathrm{Total}\;\mathrm{number}\;\mathrm{of}\;\mathrm{fish}\;\mathrm{stocked}}\times 100$

The relationships between feed supply and nutrient load, harvested fish, production cycle and net fish yield, feeding level, feed conversion efficiency, stocking density and net fish yield were estimated by the least square’s regression method, ^24^ and the figures were plotted using Microsoft Office Professional plus 2019.

## Results

### C, N and P loadings from giant gourami fish in floating cages

The C, N and P contents of the feed, fish and faeces of the giant gourami in this study are presented in Table 1. Furthermore, the estimated mass balances of C, N and P of the feed, juvenile fish, dead fish and harvested fish from the 20 floating cages are summarized in Table 2.

**Table 1.** Carbon (C), nitrogen (N) and phosphorus (P) composition (%) of the dry weight of the feed, harvested fish and faeces (±SD).

	C	N	P	H _2_O
Feed	20.23±0.10	6.02±0.29	0.71±0.03	8.75±0.01
Fish	16.56±0.13	3.01±0.07	0.40±0.03	68.90±0.77
Faeces	14.21±1.65	1.20±0.05	0.95±0.02	72.29±0.40

**Table 2.** Carbon (C), nitrogen (N) and phosphorus (P) mass balances estimated from the 20 floating cages (mean ± SD in kg/cage/cycle), and the numbers in parentheses are the range of the nutrient mass balances of C, N and P from the feed, juvenile fish, dead fish and harvested fish (kg/cage/cycle).

Nutrient	Fish feed	Juvenile fish	Mortality	Fish harvest	C, N, P loss (kg)
C	412.19±95.72 (245.27–592.23)	44.49±11.67 (23.94–63.84)	17.40±3.68 (11.84–26.23)	203.01±46.74 (11.84–26.23)	236.27±60.44 (125.65–335.95)
N	121.93±28.31 (72.55–175.19)	8.39±2.20 (4.51–12.04)	4.73±0.94 (3.28–7.27)	41.06±9.45 (25.55–60.93)	84.52±20.86 (46.74–121.06)
P	14.38±3.33 (8.55–20.66)	1.11±0.29 (0.6–1.6)	0.53±0.11 (0.36–0.80)	6.25±1.43 (3.89–9.27)	8.70±3.63 (4.73–12.40)

**Figure 1.**
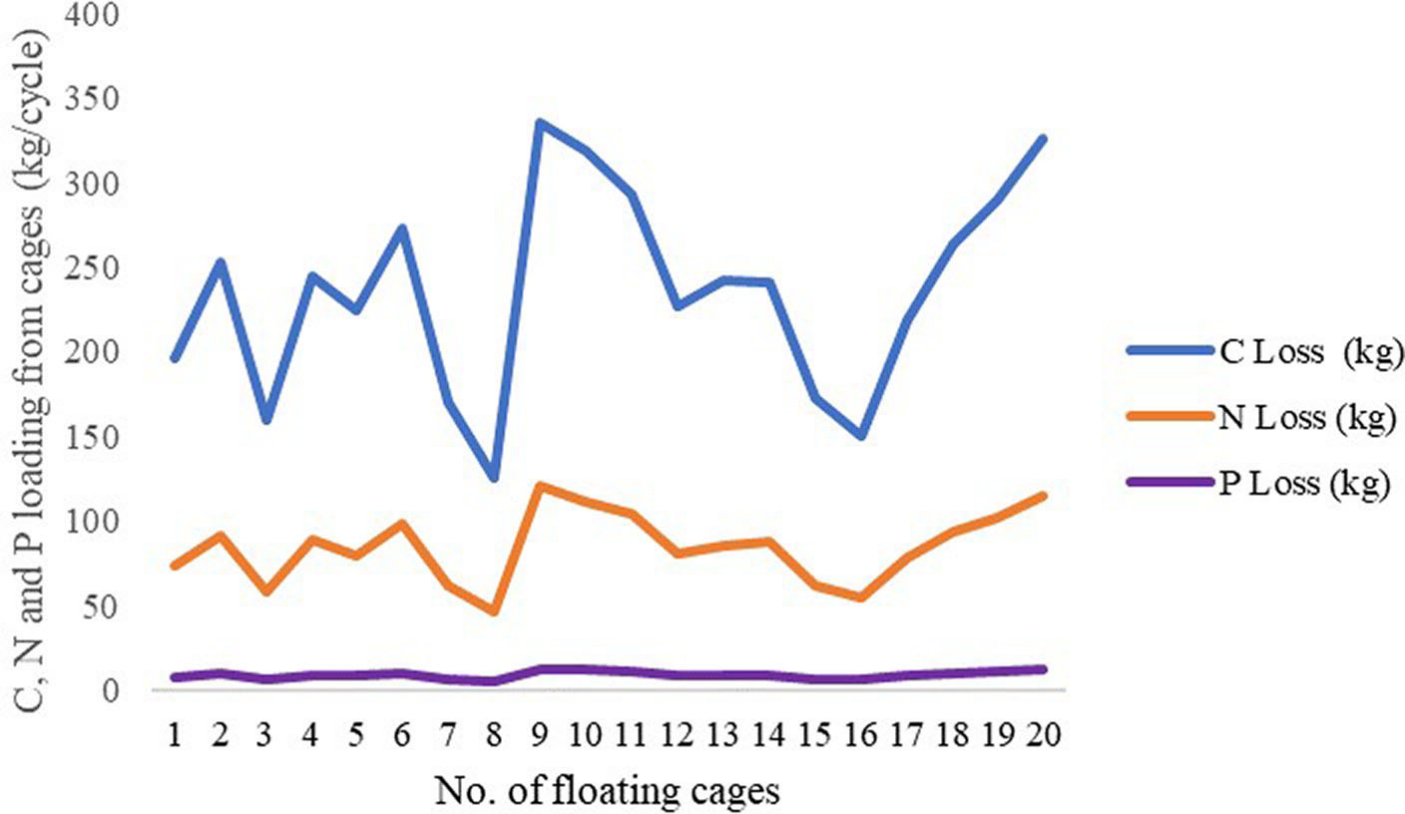
C, N and P loadings of giant gourami reared in floating cages.

**Figure 2.**
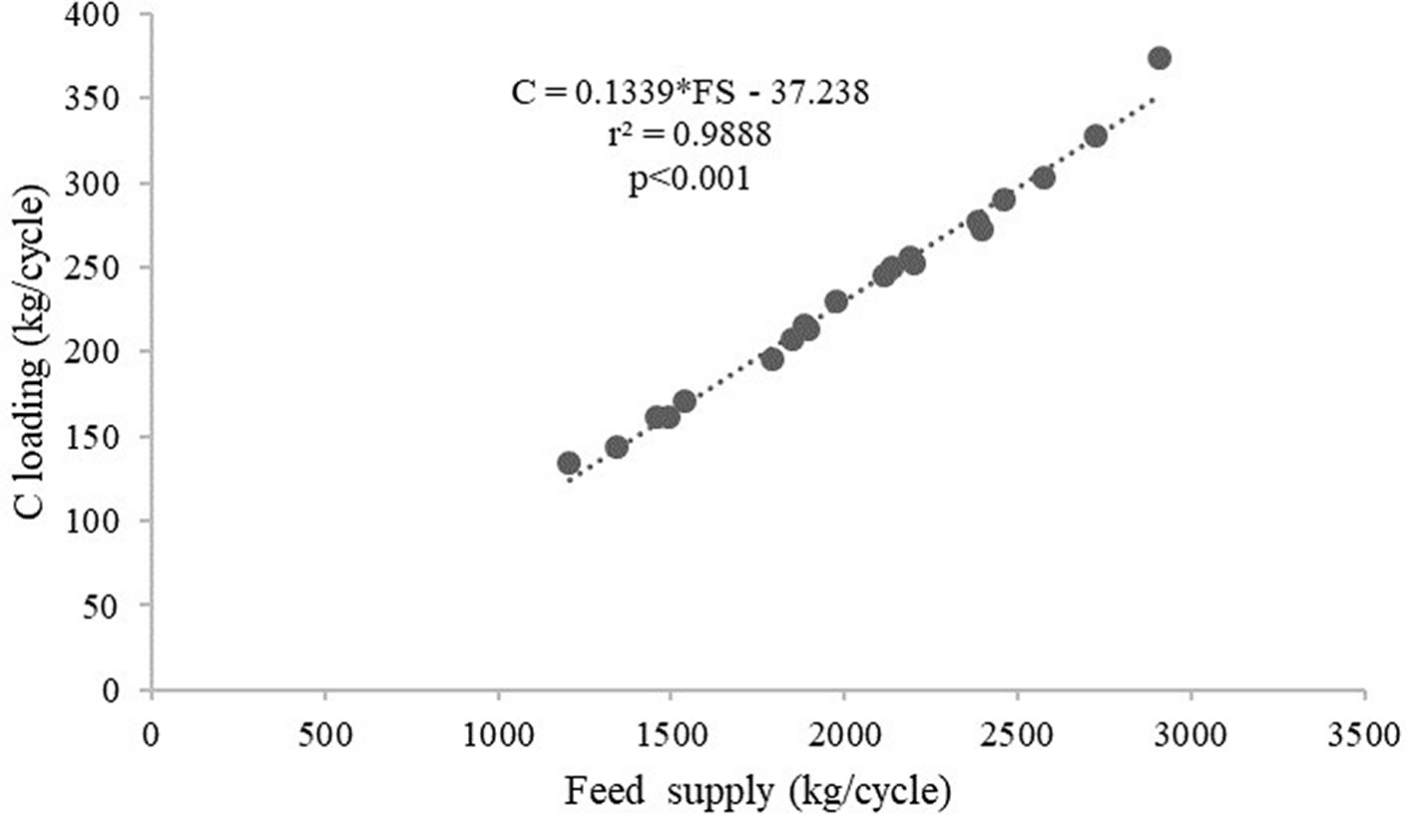
Feed supply and carbon loading relationship for giant gourami reared in floating cages.

**Figure 3.**
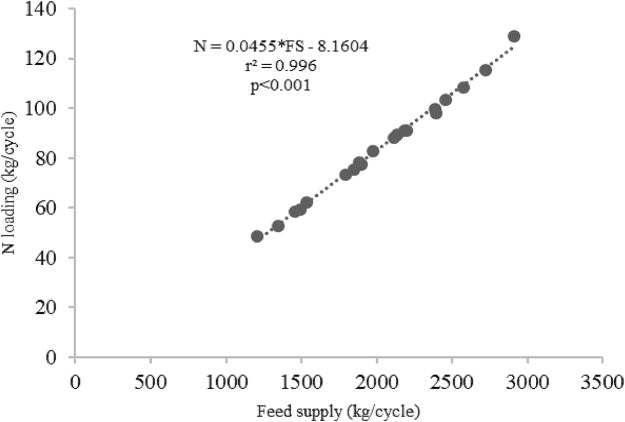
Feed supply and nitrogen loading relationship for giant gourami reared in floating cages.

**Figure 4.**
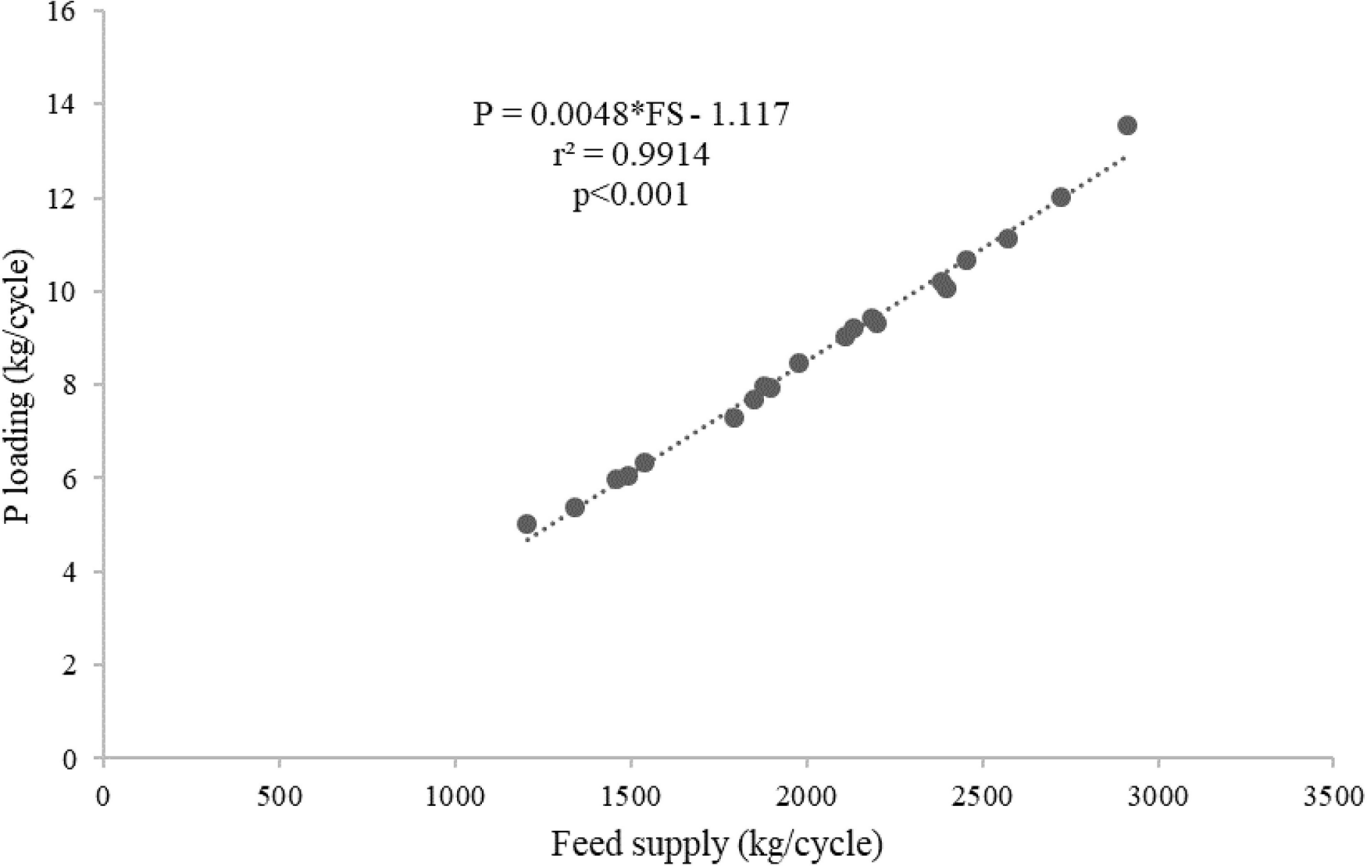
Feed supply and phosphorus loading relationship for giant gourami reared in floating cages.

**Figure 5.**
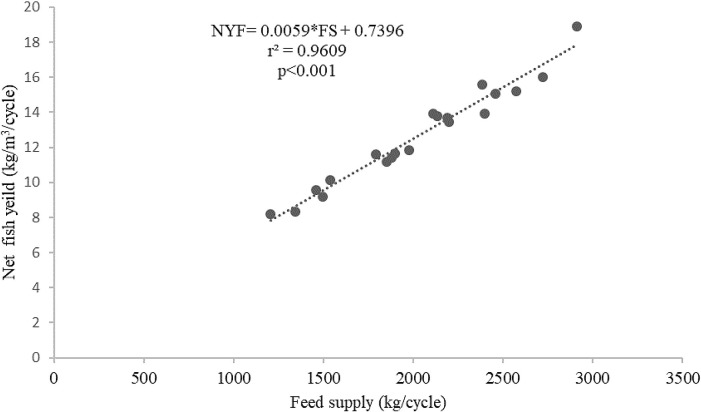
Feed supply and net fish yield (kg/m ^3^/cycle) relationship for giant gourami reared in floating cages.

Fish feed was the main factor accounting for the C, N and P nutrient loadings of the giant gourami reared in floating cages, while stocked juvenile fish and dead fish accounted for a minor amount ( Table 2). The average C, N and P loadings estimated from the floating cages were 236.27 kg/cycle, 84.52 kg/cycle and 8.70 kg/cycle, respectively, while the C, N and P loadings from each floating cage of giant gourami fish are displayed in Figure 1. Feed supply and carbon, nitrogen, and phosphorus loadings had linear relationships for the giant gourami reared in floating cages as shown by C = 0.1339 × FS − 37.238 (with *r* ^2^ = 0.988, Figure 2), N = 0.0455 × FS − 8.1604 (with *r* ^2^ = 0.996, Figure 3), and P = 0.0048 × FS − 1.117 (with *r* ^2^ = 0.991, Figure 4). The feed supply and net fish yield (kg/m ^3^/cycle) relationship for the giant gourami reared in floating cages was shown by a net fish yield=0.0059×FS+0.7396 (with *r* ^2^=0.9609, Figure 5).

### General characteristics of farms

**Figure 6.**
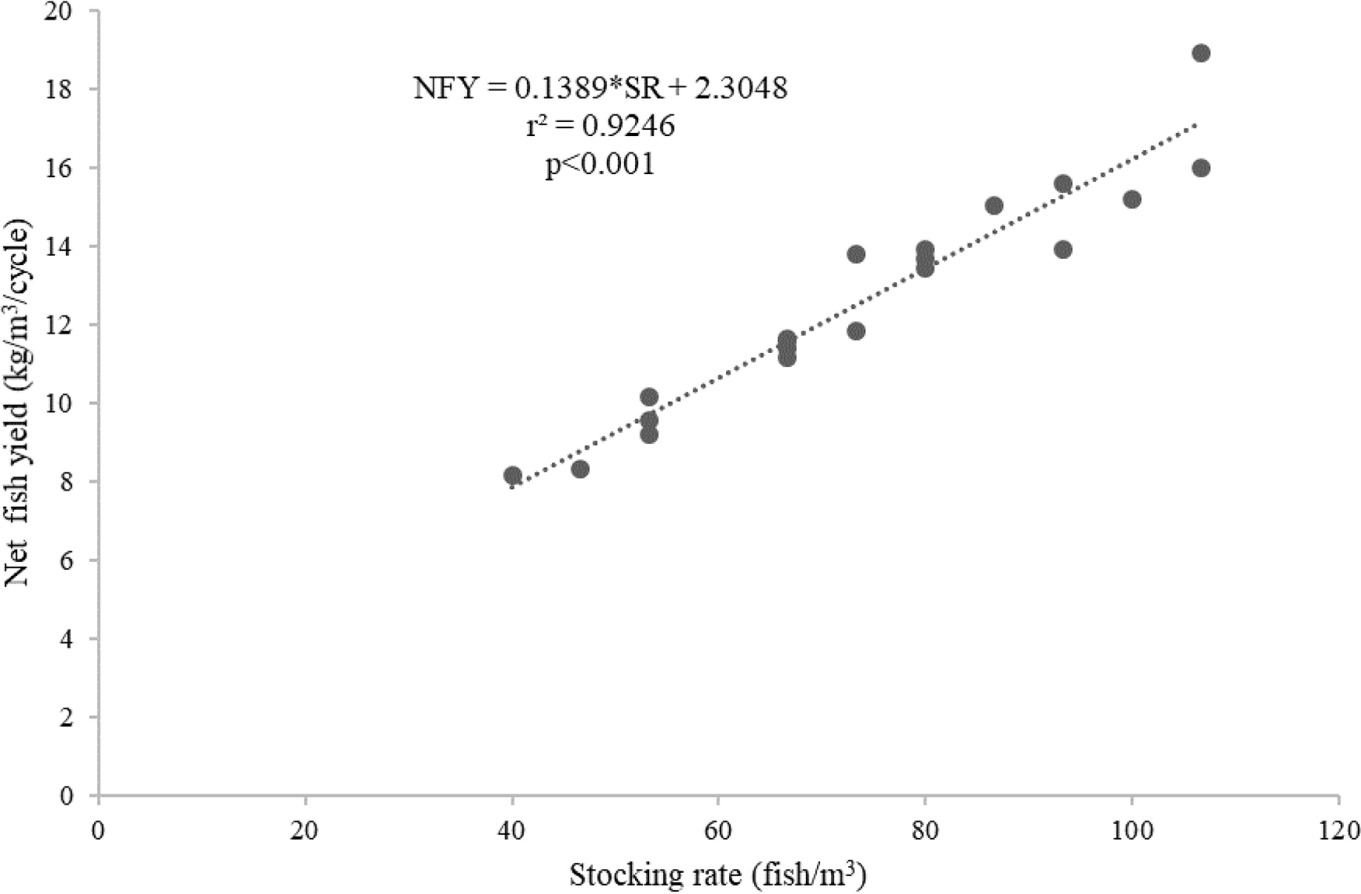
Stocking rates and net fish yield (kg/m ^3^/cycle) relationship for giant gourami reared in floating cages.

In this study, a 75 m ^3^ floating-cage capacity was used by fish farmers (5 × 5 × 3 m). The giant gourami fish stock number was between 40 individuals/m ^3^ (3000 individuals/cage) and 106 individuals/m ^3^ (8000 individuals/cage), with an average fish stock number of 75 individuals/m ^3^. A mean weight of approximately 50 g for juveniles was stocked at the beginning of culture and reared from 160 to 175 days. To maximize the growth of giant gourami, all fish farmers used commercial, floating feed pellets (30% crude protein and 5% crude lipid). Based on recorded data by the fish farmers, the fish were fed daily at 09:00–10:00 h and 16:00–17.00 h. The amount of feed provided was adjusted according to temporal changes in biomass and the growth of the fish in the floating cages during the production cycle. The results of our analysis were that their feeding levels ranged from 1.24 to 3.47% of their body mass. Harvested fish weight ranged from 225 to 290 g/fish, and the gross yield of fish was 10.4 and 24.25 kg/m ^3^/cycle, while the net fish yield was 8.17–18.92 kg/m ^3^/cycle. The giant gourami were reared in the floating cages for each production cycle of 160 to 175 days, and the specific growth rate ranged from 0.87 to 1.04%/day. The net fish yield (kg/m ^3^/cycle) in the floating cages was better predicted by the stocking rates (fish/m ^3^) ( *r* ^2^ = 0.9246, Figure 6) than by the length of the production cycles ( *r* ^2^ = 0.1875, Figure 7). In addition, the supplied feed was not strongly related to the survival of the giant gourami ( *r* ^2^ = 0.6123). On the other hand, there was a strong linear correlation between feeding levels and the net fish yield (kg/m ^3^/cycle) ( *r* ^2^ = 0.9611, Figure 8).

**Figure 7.**
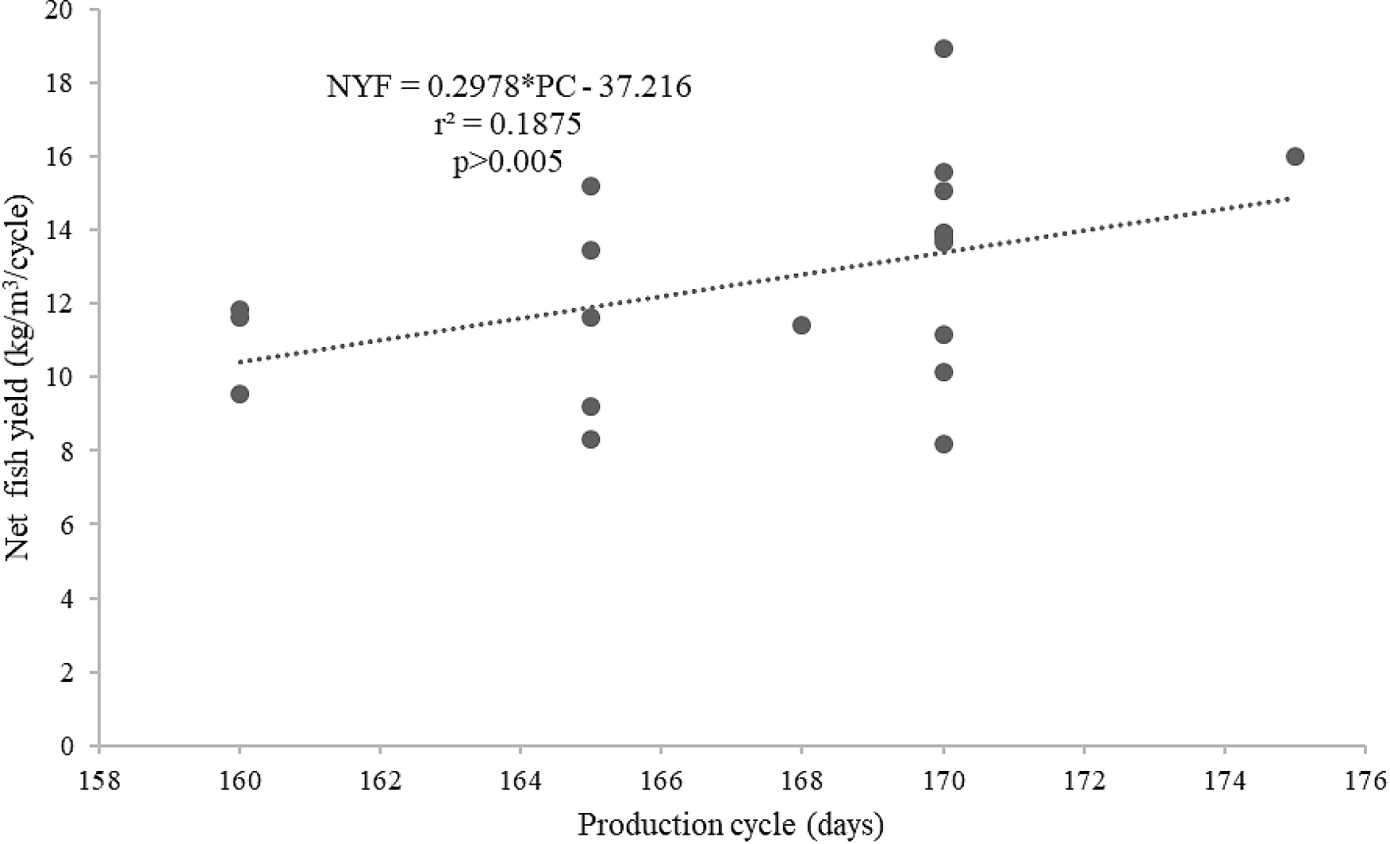
Production cycle and net fish yield (kg/m ^3^/cycle) relationship for giant gourami reared in floating cages.

**Figure 8.**
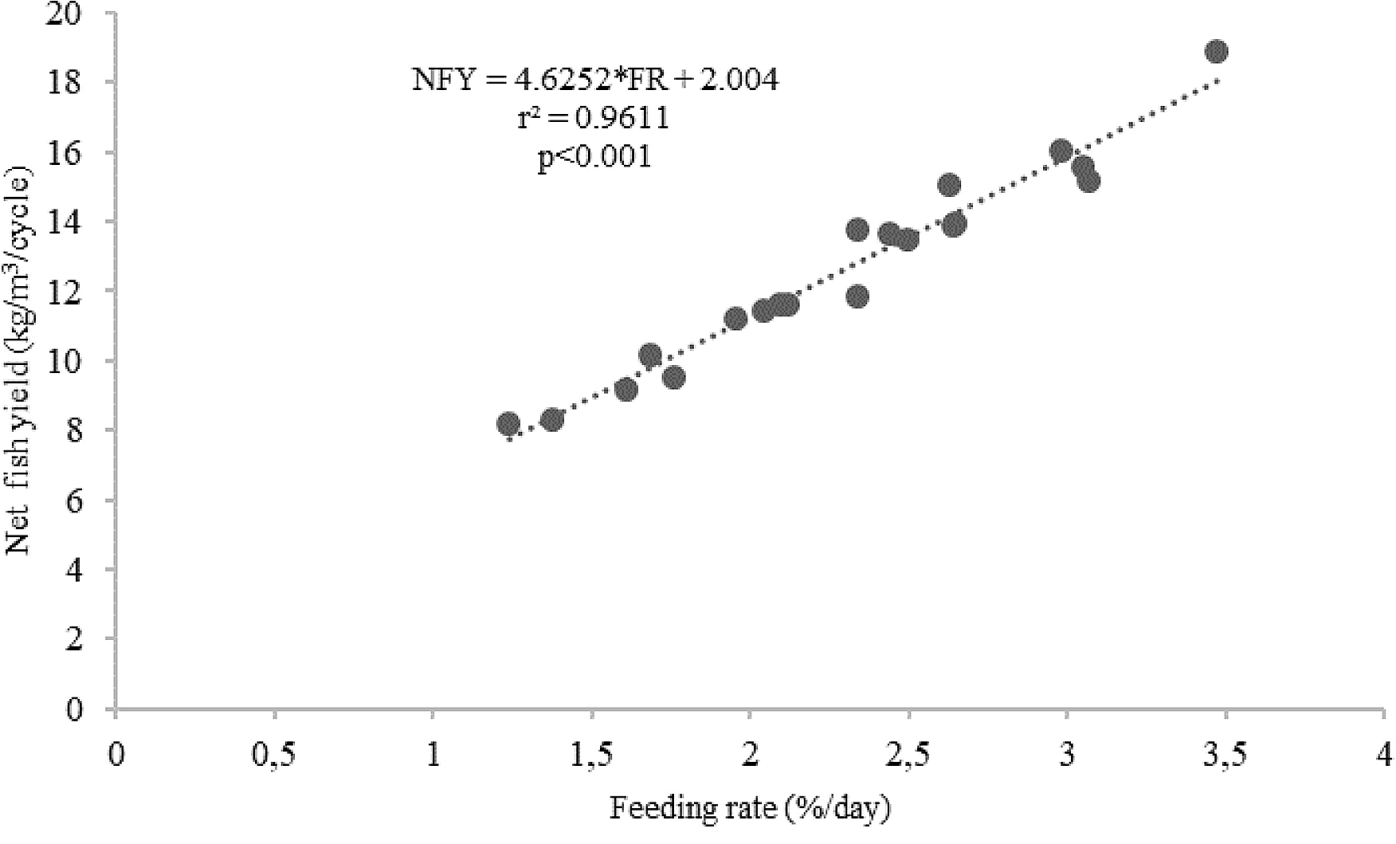
Feeding rates and net fish yield (kg/m ^3^/cycle) relationship for giant gourami reared in floating cages.

## Discussion

### Nutrient loads from floating cages

Many studies have reported that aquaculture has a negative impact on the aquatic environment, ^12^^,^^25^^–^^28^ that is generally caused by waste loads of C, N and P from supplied feed, faeces and dead fish. ^29^^–^^31^ In this study, the C, N and P loadings from the supplied feed were more predominant than those from the harvested fish, juvenile fish and dead fish because the content of C, N and P in the feed was higher than that in the harvested fish, juvenile fish and dead fish ( Table 1). In addition, the average feed conversion ratio (FCR) of the cultured giant gourami cultured was 1.65, and the feed conversion efficiency (FCE) was 0.60 (1 kg of feed fish results in 0.60 kg of fish). This result suggests that the waste load was 0.40 kg (1 kg feed−0.60 kg of fish). These FCE values were lower than those of Nile tilapia and common carp cultured in floating cages in Lake Maninjau. ^13^ Increasing amounts of C, N and P released into waterbodies from intensive aquaculture activities can cause or accelerate eutrophication in natural water systems. ^32^^–^^34^ Nevertheless, accelerated eutrophication also depends on diet composition, feed characteristics, feed intake and feed quality. ^13^^,^^35^^–^^37^ On the other hand, accelerated eutrophication in freshwater is largely determined by phosphorus. Therefore, efforts to control eutrophication in waterbodies focus mostly on phosphorus reduction. In the present study, the P load from giant gourami was 4.29 kg/tonne of feed and lower than the P load from common carp (11.45 kg/tonne of feed) and Nile tilapia (9.11 kg/tonne of feed). ^13^ Therefore, giant gourami fish farming can be considered for long-term development based on the aquaculture carrying capacity in Lake Maninjau and other regions.

Trophic food habits of fish might also affect the C, N and P was retained in the fish body because these habits are correlated with digestibility coefficients. Under natural conditions, giant gourami is an herbivorous fish. ^38^ In comparison to other fish, herbivorous fishes have more efficient digestion of feed because their extralong intestines contain special enzymes and microbes, such as cellulose enzymes and Bacteroides and Cetobacterium. ^39^^,^^40^ In the present study, the types of enzymes and microbial communities that were dominant in the giant gourami intestines are poorly understood. Regardless, herbivorous fish such as giant gourami release less N and P nutrients into waterbodies than omnivorous fish and carnivorous fish such as Nile tilapia, *Oreochromis niloticus* ^38^ and Crimson snapper, *Lutjanus erythropterus*. ^41^

In the present study, in comparison with the harvested and juvenile fish, the dead fish released only a small amount of nutrients into the waterbodies during the production cycle. Conversely, the availability of N and P in the waterbodies was significantly high after the extensive tilapia deaths due to upwelling (local namely: *tubo belerang*) and had a negative effect on the water quality of Lake Maninjau. ^33^ In contrast, giant gourami did not experience extensive fish death because this species has a labyrinth organ. Many scientists have reported that the release of significant amounts of C, N and P waste loads into waterbodies from feed and extensive fish deaths has a negative environmental impact. ^14^^,^^42^^–^^45^ In fact, feed supply and C, N and P loadings had a strong relationship with giant gourami cultured in floating cages, except in terms of fish mortality.

### General characteristics of farms

The 20,608 units of floating cages used for rearing Nile tilapia and common carp have exceeded the estimated aquaculture carrying capacity in Lake Maninjau over the past several years. ^33^ This factor has had a negative impact on the water quality of Lake Maninjau, and the net yields of Nile tilapia and common carp were 14.42 and 14.11 kg/m ^3^/cycle, respectively. ^13^^,^^46^ In contrast, poor water quality does not have a negative impact on the growth and mortality of giant gourami because this species is resistant to poor water quality. Hence, the survival of giant gourami in floating cages ranged from 86.33 to 95.27%/cycle, and the net fish yield was as high as 18.92 kg/m ^3^/cycle. In addition, the survival of giant gourami also depended on feeding level during the rearing period. Our analysis of the feeding level of giant gourami by fish farmers varied between 1.24 and 3.47%/body weight/day, and the majority of the fish farmers (80%) provided pellet feed at less than 3%/body weight/day. For giant gourami, a feeding level of 4-6%/body weight/day has been recommended. ^47^ Similarly, Skov *et al.* ^48^ concluded that biomass weight gain and the specific growth rate of Nile tilapia depend on feeding rate and the feed conversion ratio. In this study, the feeding rate and feed conversion ratio had a strong linear correlation with the net fish yield. Therefore, feeding levels played a significant role in increasing the net giant gourami yield. Many studies have reported that a lower feeding level might result in slow growth and inefficient aquaculture, whereas overfeeding may lead to feed waste, inefficiency and negative environmental impacts. ^37^^,^^49^^–^^51^

On the other hand, the length of the production cycle did not have a strong linear correlation with the net giant gourami yield (kg/m ^3^/cycle). In contrast, the stocking rate had a strong correlation with the net fish yield. In this study, the stocking rate ranged from 40 to 107 fish/m ^3^, and the majority (70%) ranged between 40 and 80 fish/m ^3^. Therefore, we recommended achieving a market size of 300 g/fish and a net fish yield (18.92 kg/m ^3^/cycle) using a stocking density of 107 fish/m ^3^ for 170 days of culture. Conversely, if the equation by Schmittou ^23^ was applied to meet the target mean weight of 300 g/fish and net fish yield at harvest of 30.93 kg/m ^3^/cycle, then we recommend using a stocking density of 106 fish/m ^3^, with a length production cycle of 170 days. Therefore, to increase production performance of giant gourami in floating cages the management strategy must be to control the optimal seed stock, fish health, feed quality, feeding level, feeding time and husbandry factors. Based on current scientific knowledge, scientists strongly advocate a combination of optimal stocking density, feeding practices, rearing techniques and eco-dam system to increase fish production performance and reduce the aquaculture waste released into waterbodies. ^41^^,^^52^^,^^53^

## Conclusion

This research analysed the carbon, nitrogen and phosphorus loadings and the farm characteristics of giant gourami reared in floating cages in Lake Maninjau. There was a strong linear relationship between feed supply and nutrient loading for the reared giant gourami. Nutrient loading from feed supply was greater than that from juvenile fish, dead fish and harvested fish. Keys to increasing the net fish yield were stocking density and feeding level. The maximum target for the net fish yield and market size was achieved for 160 days. Therefore, giant gourami cultivation is an important practice to consider continuing in Lake Maninjau in accordance with the aquaculture carrying capacity because the phosphorus released into the waterbodies was very low, and this species also has a high survival rate in floating cages.

## Data availability

### Underlying data

Fig share: Underlying data for ‘Nutrient loading and farm characteristics of giant gourami fish aquaculture systems in Lake Maninjau, Indonesia: basic knowledge of production performance’. https://doi.org/10.6084/m9.figshare.14369999

The project contains the following underlying data:

Table 1. Carbon (C), nitrogen (N) and phosphorus (P) composition (%) of the dry weight of the feed, harvested fish and faeces

Table 2. Raw data carbon loss from 20 floating cages

Table 3. Raw data nitrogen loss from 20 floating cages

Table 4. Raw data phosphorus loss from 20 floating cages

Table 5. Raw data production performance of giant gourami fish from floating cages in Lake Maninjau

Data are available under the terms of the Creative Commons Attribution 4.0 International license (CC-BY 4.0).
