# Supplementary material for: Nutrient loading and farm characteristics of giant gourami fish aquaculture systems in Lake Maninjau, Indonesia: basic knowledge of production performance
Source: F1000Res. 2021 Sep 22;10:378. Originally published 2021 May 12. [Version 2] doi: 10.12688/f1000research.52613.2 (PMC8459621; doi:10.12688/f1000research.52613.2)
Supplement: Peer review history

# Peer review: Reviewer response for version 2

**DOI:** 10.5256/f1000research.77752.r95097

**Published:** 2021-09-27

**Authors:** Zahidah Hasan

**Affiliations:** 1 Department of Fisheries. Faculty of Fisheries and Marine Science, Padjadjaran University, Jatinangor, Indonesia

The authors have revised article as suggestion. I have no further comments, this article eligible to be indexed.

Is the work clearly and accurately presented and does it cite the current literature?

Yes

If applicable, is the statistical analysis and its interpretation appropriate?

Yes

Are all the source data underlying the results available to ensure full reproducibility?

Yes

Is the study design appropriate and is the work technically sound?

Yes

Are the conclusions drawn adequately supported by the results?

Yes

Are sufficient details of methods and analysis provided to allow replication by others?

Partly

Reviewer Expertise:

I am aquaculturist majoring in water quality

I confirm that I have read this submission and believe that I have an appropriate level of expertise to confirm that it is of an acceptable scientific standard.

# Peer review: Reviewer response for version 2

**DOI:** 10.5256/f1000research.77752.r95096

**Published:** 2021-09-22

**Authors:** Christopher Mulanda Aura

**Affiliations:** 1 Department of Freshwater Department, Kenya Marine and Fisheries Research Institute, Kisumu, Kenya

The authors have incorporated my suggestions fully and I believe the paper is ready for indexing. This is a good paper for future guidance on cage culture.

Is the work clearly and accurately presented and does it cite the current literature?

Yes

If applicable, is the statistical analysis and its interpretation appropriate?

Yes

Are all the source data underlying the results available to ensure full reproducibility?

Partly

Is the study design appropriate and is the work technically sound?

Yes

Are the conclusions drawn adequately supported by the results?

Yes

Are sufficient details of methods and analysis provided to allow replication by others?

Yes

Reviewer Expertise:

Aquatic sciences

I confirm that I have read this submission and believe that I have an appropriate level of expertise to confirm that it is of an acceptable scientific standard.

# Peer review: Reviewer response for version 1

**DOI:** 10.5256/f1000research.55914.r92790

**Published:** 2021-09-14

**Authors:** Christopher Mulanda Aura

**Affiliations:** 1 Department of Freshwater Department, Kenya Marine and Fisheries Research Institute, Kisumu, Kenya

I have read through the paper and it is well written and at the same time, it will add value to cage culture knowledge on Ecosystem Approach to Aquaculture.

 Although the article is well written, there is a need to provide some data on selected physio-chemical parameters such as Oxygen, Conductivity, depth etc. that may have a role in the discussion of the results (i.e. are factors affecting nutrient loading and farm characteristics covered in terms of data to help in the discussion).

 Furthermore, it would be important if the methodology adopts a comparing scenario to offer justifiable results.

 If the above major comments are incorporated, the paper will be in good shape.

Is the work clearly and accurately presented and does it cite the current literature?

Yes

If applicable, is the statistical analysis and its interpretation appropriate?

Yes

Are all the source data underlying the results available to ensure full reproducibility?

Partly

Is the study design appropriate and is the work technically sound?

Yes

Are the conclusions drawn adequately supported by the results?

Yes

Are sufficient details of methods and analysis provided to allow replication by others?

Yes

Reviewer Expertise:

Aquatic sciences

I confirm that I have read this submission and believe that I have an appropriate level of expertise to confirm that it is of an acceptable scientific standard, however I have significant reservations, as outlined above.

# Peer review: Reviewer response for version 1

**DOI:** 10.5256/f1000research.55914.r88018

**Published:** 2021-07-23

**Authors:** Zahidah Hasan

**Affiliations:** 1 Department of Fisheries. Faculty of Fisheries and Marine Science, Padjadjaran University, Jatinangor, Indonesia

This article is clearly written. The literature cited are mostly up to date. The methods are mostly clearly written except for those related to the collection of dead fish and waste material.

 Waste material collection needs to be explained further. How long is the trap installed; is the trap removed periodically or left throughout the cultivation period? Is there also faeces in the waste material component?

Is the work clearly and accurately presented and does it cite the current literature?

Yes

If applicable, is the statistical analysis and its interpretation appropriate?

Yes

Are all the source data underlying the results available to ensure full reproducibility?

Yes

Is the study design appropriate and is the work technically sound?

Yes

Are the conclusions drawn adequately supported by the results?

Yes

Are sufficient details of methods and analysis provided to allow replication by others?

Partly

Reviewer Expertise:

I am aquaculturist majoring in water quality

I confirm that I have read this submission and believe that I have an appropriate level of expertise to confirm that it is of an acceptable scientific standard.
